# Instability of Oculomotor Control in Parkinson's Disease Without Freezing of Gait: Evidence From Reflexive and Voluntary Saccade Variability

**DOI:** 10.1111/ejn.70443

**Published:** 2026-03-22

**Authors:** Fatemeh Sadat Daeinejad, Mahmoud Saadat Foumani, Saeed Behzadipour, Laila Alibiglou

**Affiliations:** ^1^ Department of Mechanical Engineering Sharif University of Technology Tehran Iran; ^2^ Djavad Mowafaghian Research Center for Intelligent Neuro‐Rehabilitation Technologies Sharif University of Technology Tehran Iran; ^3^ Department of Physical Therapy, School of Health and Human Sciences Indiana University Indianapolis Indianapolis Indiana USA; ^4^ Stark Neurosciences Research Institute Indiana University School of Medicine Indianapolis Indiana USA

**Keywords:** antisaccade, eye movements, eye tracking, fixation, Parkinson's disease, prosaccade

## Abstract

Parkinson's disease (PD) is associated with alterations in both voluntary and reflexive eye movements; however, the characteristics of oculomotor variability across task contexts remain incompletely understood. This study investigated prosaccade, antisaccade and fixation parameters in 15 individuals with early‐ to midstage PD, assessed in the on‐medication state and 15 age‐ and sex‐matched neurologically healthy controls. Eye movements were recorded during structured saccadic tasks and during free‐viewing of dynamic video stimuli using high‐resolution binocular eye tracking. Compared with controls, participants with PD exhibited significantly higher prosaccade error rates and an increased peak velocity‐to‐amplitude ratio. Trial‐to‐trial variability, quantified using coefficients of variation, was consistently elevated in the PD group across multiple saccade parameters. During the video‐viewing condition, changes in saccade metrics following video exposure were observed in the control group but not in the PD group, whereas fixation‐based measures did not reliably differentiate groups. Together, these findings indicate that increased variability and reduced consistency of saccadic execution are prominent features of oculomotor control in PD without freezing of gait, particularly during reflexive saccade tasks. The results underscore the value of variability‐based analyses for probing sensorimotor control in PD and motivate future work to examine their task dependence, longitudinal stability and relevance across disease stages.

## Graphical abstract

This study found that people with Parkinson's disease without freezing of gait show higher error rate and peak velocity to amplitude ratio during prosaccade task compared to controls. Also, Parkinson patients show more variation in gain, peak velocity, peak acceleration and peak velocity to amplitude ratio compared to control group. These findings suggest that prosaccade task is critical for understanding eye movement abnormalities in Parkinson, especially Parkinson patients without freezing of gait.

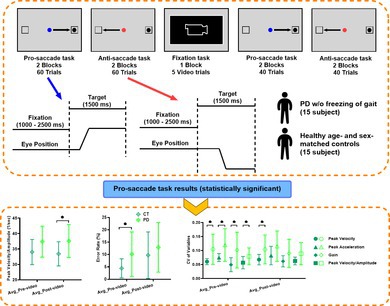

**Abbreviations**
CT: neurologically healthy control individuals
CV: coefficient of variation
FOG: freezing of gait
FOGQ: the Freezing of Gait Questionnaire
ICC: intraclass correlation coefficient
IQR: interquartile range
IRB: the Institutional Review Board
I‐VT: velocity‐threshold identification
ON‐Med: on‐medication state
PD: Parkinson's disease
UPDRS: Unified Parkinson Disease Rating Scale

## 1 Introduction

Parkinson's disease (PD) is the second most prevalent neurodegenerative disorder, following Alzheimer's disease. It is characterized by a dopamine deficiency which leads to a range of movement‐related problems including bradykinesia, rigidity and tremor (de Lau and Breteler 2006; Elbaz et al. 2016). Considering the rapidly increasing population of individuals affected by PD, substantial efforts are being directed toward advancing a comprehensive understanding of the diverse characteristics of PD.

Eye movement abnormalities are one category of PD characteristics. These abnormalities can manifest as impairments in various types of eye movements and pupil size (Armstrong 2011; Pretegiani and Optican 2017; Tsitsi et al. 2021). Recent advancements in eye‐tracking technology have enabled relatively precise and non‐invasive measurement of eye movement signals, facilitating the study of brain function (Harezlak 2017). Although numerous studies have demonstrated that eye movements are altered in Parkinson's disease, further work is needed to clarify how different features of oculomotor control vary across task contexts and disease stages (Antoniades and Spering 2023).

Fixation and saccades are two fundamental types of eye movements that have been extensively studied in individuals with PD. Fixation refers to the maintenance of gaze on a single location, during which the eyes remain relatively stable to allow for detailed visual processing. Saccades are rapid, ballistic eye movements that move the fovea from one point of interest to another between fixations (Kok and Jarodzka 2017). Two commonly used paradigms for measuring saccades are the antisaccade (or voluntary saccade), a saccadic eye movement made in the opposite direction of a visual target, and the prosaccade (or reflexive saccade), a rapid eye movement made toward a visual target (Antoniades et al. 2013; Stuart et al. 2019).

In the context of voluntary saccades (antisaccades), patients with PD have demonstrated higher error rates and longer latencies compared to age‐matched neurologically healthy controls (Briand et al. 1999; Chan et al. 2005; van Stockum et al. 2012). However, the findings regarding reflexive saccades (prosaccades) have been inconsistent. Some studies indicate that individuals with PD exhibit impairments in reflexive saccades, characterized by significantly shorter and slower saccadic movements, reflecting reduced average velocity when compared to controls (Stuart et al. 2019). Conversely, other studies have shown that reflexive saccades tend to be normal in people with PD (Briand et al. 1999; Chan et al. 2005; van Stockum et al. 2012; Gorges et al. 2014). Although previous studies have suggested that freezing of gait (FOG) may contribute to this inconsistency (Nemanich and Earhart 2016), our study specifically excluded participants with FOG to minimize confounding factors associated with more advanced or multifactorial pathology. FOG is defined by brief, episodic pauses in locomotion and is linked to additional cognitive and motor deficits (Hely et al. 2008; Shine et al. 2013; Amboni et al. 2015; Forsaa et al. 2015), which could influence saccadic performance. By concentrating on individuals without FOG, we aimed to evaluate oculomotor function in early to midstage PD while avoiding the additional variability presented by this phenomenon. While some studies have examined differences in saccade characteristics between PD patients with and without FOG (Nemanich and Earhart 2016; Walton et al. 2015; Wu et al. 2020; Graham et al. 2023), few have focused on fixation metrics in PD without FOG. Notably, Nemanich and Earhart (2016) reported significant differences in antisaccade error rate and the coefficient of variation for peak velocity between PD patients without FOG and healthy controls. However, fixation metrics beyond duration remain largely underexplored in this subgroup.

Considering that FOG typically emerges later in the progression of Parkinson's disease, focusing on individuals with PD without FOG allows characterization of oculomotor control during earlier disease stages, before the development of prominent gait disturbances. In this context, examining trial‐to‐trial variability, rather than relying solely on mean performance, may provide additional insight into disruptions in motor planning and sensorimotor integration within the neural circuits governing eye movements in PD. In the present study, we investigated eye movement parameters during fixation, reflexive (prosaccade) and voluntary (antisaccade) tasks in individuals with PD without FOG compared to age‐ and sex‐matched neurologically healthy controls. We assessed a variety of eye movement parameters, including latency, peak velocity, acceleration, gain, the velocity to amplitude ratio, error rates and trial‐to‐trial variability (coefficient of variation) across these tasks. Participants also completed a naturalistic video‐viewing task, which allowed us to evaluate the influence of contextual visual input on saccade performance. During these fixation tasks, we analysed the number of fixations, fixation duration and fixation dispersion as participants viewed five videos depicting distinct real‐world scenarios. By comparing pre‐ and postvideo saccade parameters within and between groups, we aimed to examine whether saccade parameters were modulated by visual context in individuals with PD compared with controls.

We hypothesized that individuals with PD would demonstrate increased variability and error rates in prosaccades, differences in fixation behaviour and context‐dependent modulation of saccade parameters, even while on medication. Participants with PD were assessed in their on‐medication state to accurately reflect real‐world clinical functioning and to minimize the confounding effects of medication withdrawal, which can introduce further motor and cognitive variability. Previous research has indicated that dopaminergic therapy can affect saccadic behaviour, including aspects such as gain, velocity and variability (Hood et al. 2007; Reilly et al. 2008; Lu et al. 2019; Branyiczky et al. 2025). We aimed to characterize fixation and saccade behaviour across reflexive and voluntary tasks in individuals with PD without FOG, with particular emphasis on trial‐to‐trial variability and task dependence. Rather than identifying diagnostic markers, this approach seeks to describe features of oculomotor control that may reflect altered sensorimotor processing in PD and motivate future hypothesis‐driven and longitudinal investigations.

## 2 Methods

### 2.1 Participants

Fifteen individuals with idiopathic Parkinson's disease (PD) (four female and 11 male) and 15 age‐ and sex‐matched neurologically healthy controls (CT) participated in this study. The sample size was determined based on previous studies evaluating *saccadic* eye *movements* in PD patients versus matched controls (Nemanich and Earhart 2016; Walton et al. 2015).

PD participants were diagnosed according to the UK Parkinson's Disease Society Brain Bank criteria and evaluated by a neurologist in Movement Disorders. Clinical severity was assessed using the Movement Disorder Society‐Unified Parkinson's Disease Scale (MDS‐UPDRS) Part III *(*Motor Examination). The inclusion criteria for all PD participants required Hoehn and Yahr Stage II–III (Hoehn and Yahr 1967), the ability to follow and understand instructions, as the absence of FOG (as assessed by the Freezing of Gait Questionnaire (FOGQ) and specifically Question 3 (Giladi et al. 2000)) and normal or corrected‐to‐normal vision. Exclusion criteria included other neurological disorders, brain surgery or deep brain stimulation, significant resting tremor (UPDRS items 20 or 21 > 2), or recent changes in dopaminergic medication (within the last month).

All participants were tested in the morning while PD patients were in their on‐medication state (approximately 2 h after L‐DOPA administration). Although controlling for medication dose was beyond this study's scope, we acknowledge its potential influence on saccade dynamics and will discuss this as a limitation. One participant with PD had a history of a retinal tear; analyses with and without this participant yielded comparable group‐level results. The same exclusion criteria applied to the PD group were also applied to the neurologically healthy controls. This study was approved by the local Institutional Review Board (IRB) and conducted in accordance with the Declaration of Helsinki. All participants provided informed, written consent before participation.

### 2.2 Experimental Protocol

Participants performed a battery of eye movement tasks designed to evaluate both structured saccadic behaviour and visual attention under naturalistic conditions. The experimental session consisted of five sequential blocks: a baseline block, a prevideo block, naturalistic video‐viewing block, a postvideo block and a retention block. The protocol followed best practices for oculomotor testing in individuals with neurological conditions (Antoniades et al. 2013).

Eye movements were recorded binocularly using an EyeLink 1000 Plus system (SR Research Ltd., Ottawa, Canada), sampled at 1000 Hz. Participants were seated approximately 70 cm from a 24‐in. LCD monitor, which was adjusted to eye level. A chin rest was used to minimize head movements. Calibration was performed individually for each participant using a 9‐point grid (biquadratic with corner correction) (Stampe 1993). Raw eye position data were recorded for 1500 ms following target onset.

**FIGURE 1:**
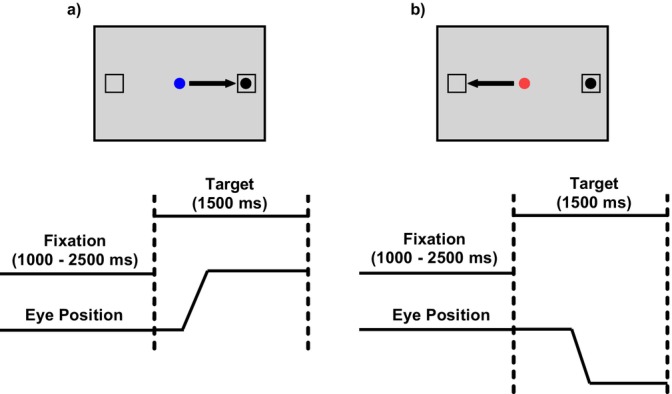
Saccadic tasks. (a) prosaccade and (b) antisaccade.

The experimental session began with a baseline period consisting of two blocks: one containing 40 prosaccade trials and one containing 40 antisaccade trials. In both blocks, peripheral targets were randomly presented at eccentricities of 5°, 10° and 15°. Each of the three subsequent saccadic testing blocks (prevideo, postvideo and retention) included 40 trials with a fixed target eccentricity of 10°, equally divided between prosaccade and antisaccade trials. All trials followed the same structure: a central fixation circle (blue for prosaccade and red for antisaccade) appeared on a grey background, followed after a randomized delay (1000–2500 ms) by the appearance of a peripheral black target (0.6° diameter) on either the left or right side (Figure 1). Participants were instructed to execute the appropriate saccadic response as quickly and accurately as possible. The target remained on the screen for 1500 ms, followed by a 500‐ms intertrial interval. Prior to testing, each participant completed three practice trials of each saccade type.

**FIGURE 2:**
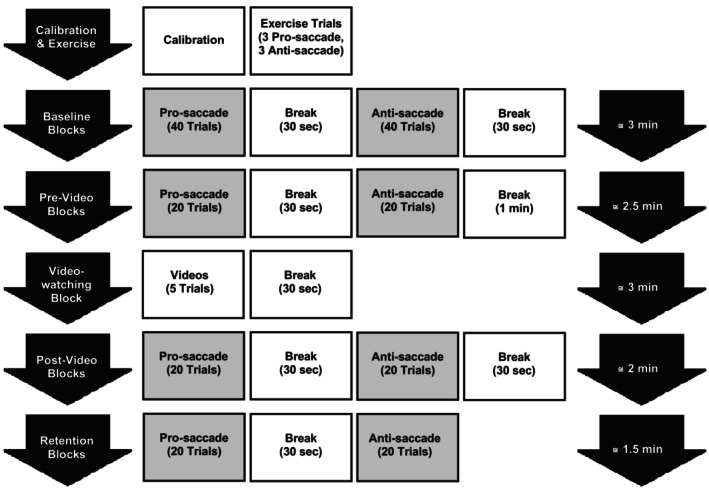
Experimental protocol. Experiments started with calibration, and next were six exercise trials (three prosaccade trials and three antisaccade trials). Then, baseline blocks began, which included two sets of trials: one set of 40 prosaccades trials and one set of 40 antisaccades trials. After the baseline blocks, the experiment included four blocks (prevideo, video‐watching, postvideo and retention). Each prevideo, postvideo and retention block included 40 trials (20 prosaccades and 20 antisaccades trials). During the video‐watching block, five videos of different environments and situations were shown to subjects. A break of 30 s was provided between each block and before the video‐watching block, an extra 30 s break (1 min in total) was considered.

Fixation patterns are typically examined during tasks such as reading, scene viewing or video observation (Herten et al. 2017). In the current study, the naturalistic video‐viewing block involved passive observation of five short video clips (each approximately 30 s in duration) depicting real‐world environments known to challenge visuomotor coordination and induce psychological stress in individuals with Parkinson's disease. These scenarios included walking across a narrow bridge over a deep hole, riding a fast‐turning roller coaster, boarding a crowded train, navigating a narrow corridor lined with doors and passing through an automatic door just before it closed. Although none of the participants in this study exhibited FOG, such environments have been widely reported by individuals with PD as provoking disorientation, anxiety or freezing episodes. Accordingly, this block was designed to simulate visually complex, stress‐inducing conditions to examine whether exposure to such contexts modulates subsequent saccadic performance or visual attention. This approach was intended to enhance ecological validity of the protocol and investigate the potential sensitivity of oculomotor behaviour to environmental context. Each data collection session included 200 saccade trials and five video trials. The timeline of the experimental protocol is depicted in Figure 2.

To minimize fatigue and ensure data reliability, short breaks of 30 s were provided between each block, with an additional 30‐s break before the video‐watching block. The total session duration, including calibration and practice, was kept under 40 min (Antoniades et al. 2013). All task logic and stimuli presentation were implemented using Experiment Builder software (SR Research, Ottawa, Canada).

### 2.3 Data Analysis

Eye movement data from saccade blocks were initially processed using Data Viewer software (SR Research Ltd., Ottawa, Canada), which extracted the raw eye position data. Subsequent data processing and quantitative analysis were conducted using a custom MATLAB script (R2017b, The Mathworks Inc., Natick, MA), following a standardized protocol for saccade analysis (Nij Bijvank et al. 2018). To reduce noise, raw gaze data were filtered using a maximally flat, second‐order low‐pass filter with a 100‐Hz cut‐off (10% of the sampling frequency), followed by convolution with a nine‐sample edge‐enhancing smoothing kernel. Blink‐related artefacts were removed by discarding samples from 100 ms before to 250 ms after each blink.

Velocity and acceleration signals were derived through temporal differentiation of position and velocity data, respectively. Saccades were detected using an adaptive acceleration threshold based on the 99% confidence interval of the acceleration distribution (samples exceeding 2.57 standard deviations above the mean). This individualized thresholding approach accommodated interparticipant and trial‐level variability in signal quality.

A detected saccade was considered valid if its amplitude was ≥0.15°, and duration was at least 8 ms. Within each saccadic interval (defined as consecutive saccades separated by intersaccadic intervals < 20 ms), the saccade with the highest peak velocity was classified as the main saccade. This approach was chosen to improve detection robustness in trials consisting small corrective saccades or micromovements and was applied consistently across all participants and conditions.

In pro‐ and antisaccade trials, a saccade was considered as correct if it was initiated between 50 and 1000 ms after target onset, originated within 2.5° of visual angle from the central fixation point, and was directed toward the appropriate spatial location (i.e., toward the target for prosaccades and away from the target for antisaccades). A 50‐ms lower bound cut‐off was applied to conservatively exclude anticipatory saccades, while retaining early visually guided responses, consistent with the analytic framework described by Nij Bijvank et al. (2018). This criterion was applied uniformly across all participants and conditions.

Amplitude thresholds for error classification were based on task‐specific performance criteria. For prosaccade trials, a response was classified as an error if no saccade was detected or if the amplitude was less than 50% of the expected target distance (e.g., < 7.5°, < 5° or < 2.5° for 15°, 10° and 5° targets, respectively). For antisaccade trials, responses were considered errors if they were directed toward the stimulus or had an amplitude < 2°. These cut‐offs were selected to balance sensitivity and specificity in detecting true task failures, particularly in a clinical sample prone to variability. While we recognize that these criteria may differ from those used in other studies, they are justified given the task demands and signal quality in this cohort.

The following parameters were computed for all valid pro‐ and antisaccade trials: peak velocity, peak acceleration, gain (saccade amplitude relative to mean fixation amplitude after any corrective saccades), latency (the time from stimulus onset to saccade onset) and the ratio of peak velocity‐to‐amplitude. To capture intertrial variability, the coefficient of variation (CV) was calculated for each parameter (Nemanich and Earhart 2016; Nij Bijvank et al. 2018; Archibald et al. 2013). This approach to calculating gain was chosen to reduce the impact of system inaccuracies in measuring absolute eye position (Nij Bijvank et al. 2018). Depending on data distribution, parameter central tendencies were computed using the mean and standard deviation (for normally distributed variables) or the median and interquartile range (IQR) for nonnormally distributed ones. CVs were calculated as the SD‐to‐mean ratio for normal data and IQR‐to‐median ratio for nonnormal data. Only binocular saccades were analysed; trials were excluded if the left and right eye differed by more than 2° in amplitude or more than 20 ms in onset time.

The pro‐/antisaccade error rate was defined as the ratio of incorrect trials to the total number of valid trials within each block. Trials with no valid saccade in either eye, or with large binocular discrepancies, were excluded from further analysis.

For the naturalistic video‐viewing block, fixation analysis was conducted using the Data Viewer software's fixation reports, which are based on the EyeLink system's real‐time I‐VT (velocity‐threshold identification) classification. Although there is no universal standard for fixation detection during free‐viewing of dynamic scenes, the I‐VT algorithm has shown reliable performance in naturalistic settings (Munn et al. 2008; Andersson et al. 2017). Given that we aimed to quantify broad fixation patterns rather than smooth pursuit or microsaccades, this classification approach was deemed appropriate. We used the system's default velocity (30°/s) and acceleration (8000°/s^2^) thresholds and a minimum fixation duration of 200 ms, consistent with prior studies (Munn et al. 2008).

Fixation parameters included mean fixation duration, number of fixations and fixation dispersion, the latter defined as the root mean square of Euclidean distances between each fixation and the mean fixation position. This measure reflects the spatial extent of gaze exploration across the visual field (Archibald et al. 2013; Zhang et al. 2020).

### 2.4 Statistical Analysis

All statistical analyses were conducted using MATLAB (R2017b, The Mathworks Inc., Natick, MA) and SPSS (v26, IBM Corp, Armonk, NY). Group‐level demographic variables were compared using independent‐samples *t*‐tests for normally distributed data and Mann–Whitney *U* tests for nonnormally distributed data.

To assess within‐group differences in pro‐ and antisaccade error rates and parameter variability, paired‐samples *t*‐test, Wilcoxon signed‐rank tests or paired‐samples sign tests were used depending on the distributional characteristics of the data (i.e., normal vs. nonnormal and symmetrical vs. nonsymmetrical).

Test–retest reliability of saccade parameters was evaluated using intraclass correlation coefficients (ICCs), calculated via a two‐way mixed‐effects ANOVA model (average measures and absolute agreement). Data from the baseline and prevideo blocks were considered one set (preintervention), and postvideo and retention blocks were treated as the second set (postintervention). ICC values were interpreted according to established criteria: excellent (ICC > 0.90), good (0.75–0.90), moderate (0.50–0.75) and poor (< 0.50) (Koo and Li 2016). Given that most parameters showed acceptable reliability (ICC > 0.75), we averaged the baseline and prevideo blocks, as well as the postvideo and retention blocks, to increase statistical power and reduce trial‐wise noise. While this approach enhances robustness, we acknowledge that averaging across timepoints may obscure block‐specific effects. To mitigate this, we also conducted supplementary comparisons between baseline and prevideo and between postvideo and retention, to confirm that any changes were specifically attributable to the video‐viewing intervention rather than time alone. Between‐group comparisons of primary saccade parameters and their coefficients of variation (CVs) were performed using independent‐samples *t*‐tests or Mann–Whitney *U* tests, depending on data distribution.

Within‐group comparisons of task‐specific effects (e.g., pro‐ vs. antisaccade performance) and video‐induced changes (pre/post) were analysed using paired‐samples *t*‐tests or nonparametric equivalents. For analyses involving the naturalistic video‐viewing block, independent‐samples *t*‐tests or Mann–Whitney *U* tests were used to compare fixation‐related parameters (mean fixation duration, number of fixations and fixation dispersion) across groups for each video and for the average across all videos.

Within‐group comparisons across the five video stimuli were conducted using repeated‐measures ANOVA for normally distributed data (with no significant outliers and sphericity assumptions met) or the Friedman test for nonnormally distributed data. Significant Friedman tests were followed by Wilcoxon signed‐rank or paired‐samples sign tests for post hoc comparisons.

All tests were two‐tailed with a significance threshold of *α* = 0.05. When multiple comparisons were performed, Bonferroni correction was applied to adjust for the risk of Type I error inflation. In addition to reporting *p*‐values, we also report effect sizes (Cohen's *d* or rank biserial correlation, as appropriate) and 95% confidence intervals to support interpretation of both significant and nonsignificant findings.

## 3 Results

Demographics and clinical characteristics of two groups are summarized in Table 1. There was no significant difference in age between the two groups (*t*(23.318) = 0.451, *p* = 0.656).

**TABLE 1:** Demographic and clinical characteristics of study participants.

	Control group	PD group
Number of subjects	15	15
Age	59.40 ± 8.28	60.53 ± 5.11
Gender (male/female)	11/4	11/4
UPDRS Score (Part III)	—	34.40 ± 9.14
Hoehn and Yahr	—	2.40 ± 0.51
Years since diagnosis	—	4.80 ± 2.21

To evaluate the test–retest reliability and measurement stability, ICCs were calculated for all saccade parameters. In the prosaccade trials, both groups demonstrated excellent reliability for peak velocity, peak acceleration, latency and the peak velocity to amplitude ratio and good reliability for gain. In antisaccade trials, ICCs were excellent for all parameters in both groups except latency in the control group, which showed good reliability. These values are comparable to, or higher than, those previously reported in the neurologically healthy individuals (Nij Bijvank et al. 2018).

### 3.1 Group Differences in Saccade Metrics

#### 3.1.1 Error Rates

**FIGURE 3:**
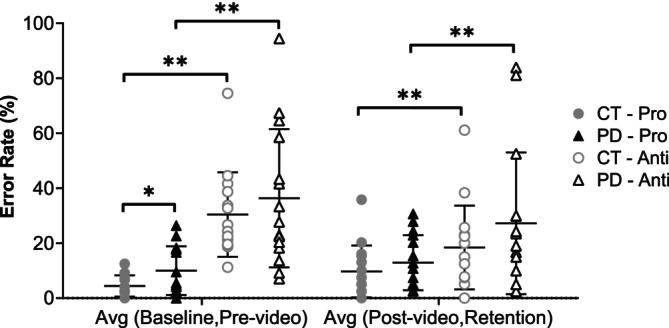
Pro‐/antisaccade error rates in both groups. Significant higher prosaccade error rate for PD group compared to CT group in the average of baseline and prevideo blocks (*p* = 0.038). Antisaccade error rate was significantly higher than prosaccade error rate in all comparisons of both CT and PD groups (*p* ≤ 0.007 and *p* ≤ 0.022). *Significant difference between two groups. **Significant difference between two tasks.

As shown in Figure 3, the PD group exhibited higher prosaccade error rates than controls. These differences reached statistical significance only in the combined baseline and prevideo blocks (*t*(19.088) = 2.236, *p* = 0.038, Hedges' g = 0.77, 95% CI [0.03, 1.51]). Antisaccade error rates were also higher in the PD group, but group differences were not significant.

#### 3.1.2 Peak Velocity/Amplitude Ratio

**FIGURE 4:**
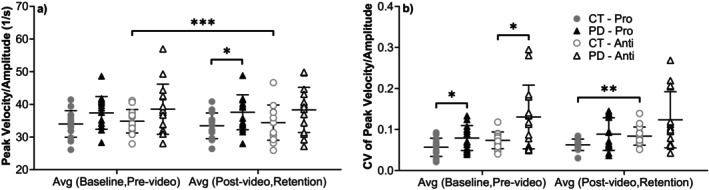
(a) Peak velocity/amplitude. PD group showed higher values, with significant or near‐significant differences compared to CT group in the average of postvideo and retention blocks (*p* = 0.025) and the average of baseline and prevideo blocks (*p* = 0.059) for prosaccade task. In antisaccade task of CT group, there were significant differences between the average of baseline and prevideo blocks and the average of postvideo and retention blocks (*p* = 0.023). (b) CV of peak velocity/amplitude. PD group showed higher values, with significant differences compared to CT group in both prosaccade and antisaccade tasks of the average of baseline and prevideo blocks (*p* = 0.027 and *p* = 0.046). Comparing two tasks, there were significant difference in the average of postvideo and retention blocks (*p* = 0.005) of CT group. *Significant difference between two groups. **Significant difference between two tasks. ***Significant difference before and after video‐watching block.

Peak velocity/amplitude values (Figure 4) were generally higher in PD than controls in both tasks. In the prosaccade condition, the difference was significant in the average of postvideo and retention blocks (*t*(27) = 2.372, *p* = 0.025, Hedges' g = 0.83, 95% CI [0.07, 1.59]) and marginal in the average of baseline and prevideo blocks (*t*(27) = 1.975, *p* = 0.059, Hedges' g = 0.69, 95% CI [−0.06, 1.44]). No significant group differences were observed for antisaccade trials. The CV of peak velocity/amplitude was significantly higher in PD group for the average of baseline and prevideo blocks for both the prosaccade (*t*(28) = 2.327, *p* = 0.027, Hedges' g = 0.79, 95% CI [0.04, 1.53]) and antisaccade (U = 54, *p* = 0.046, $r_{\mathrm{rb}}$ = −0.446) tasks.

#### 3.1.3 Gain:

**FIGURE 5:**
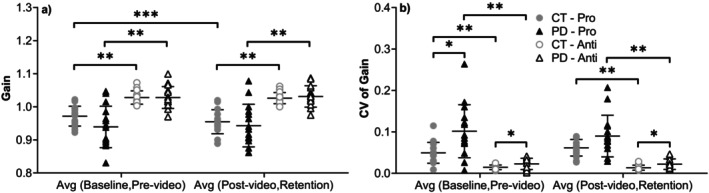
(a) Gain. There were no significant differences between the two groups. Comparing two tasks, there was a significant difference in all comparisons of both groups (*p* < 0.001). In the prosaccade task of the CT group, there were significant differences between the average of baseline and prevideo blocks and the average of postvideo and retention blocks (*p* = 0.007). (b) CV of gain. The CV in the PD group was significantly higher than the CT group in the average of baseline and prevideo blocks (*p* = 0.009) of the prosaccade task. In the antisaccade task, the differences between the two groups were significant in both the average of baseline and prevideo blocks (*p* = 0.043) and the average of postvideo and retention blocks (*p* = 0.045). Comparing two tasks, there was a significant difference in all comparisons of the CT and PD groups (*p* ≤ 0.001). *Significant difference between two groups. ** Significant difference between two tasks. *** Significant difference before and after video‐watching block.

Gain values (Figure 5) did not differ significantly between groups in the prosaccade task, although controls tended to show slightly higher gains.

The CV of gain was consistently higher in PD. This difference reached significance in the average of baseline and prevideo blocks for the prosaccade task (*t*(18.19) = 2.947, *p* = 0.009, Hedges' g = 1.03, 95% CI [0.26, 1.79]) and was marginal in the average of postvideo and retention blocks (*t*(18.305) = 2.024, *p* = 0.058, Hedges' g = 0.69, 95% CI [−0.05, 1.43]). For the antisaccade task, gain variability was significantly higher in PD in both the average of baseline and prevideo blocks (*t*(18.660) = 2.176, *p* = 0.043, Hedges' g = 0.74, 95% CI [0.00, 1.48]) and the average of postvideo and retention blocks (U = 64, *p* = 0.045, $r_{\mathrm{rb}}$ = −0.431).

#### 3.1.4 Latency

**FIGURE 6:**
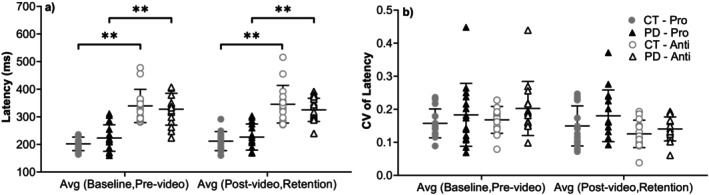
(a) Latency. There were no significant differences between two groups. There were significant differences between prosaccade and antisaccade tasks in all comparisons of both groups (*p* < 0.001). (b) CV of latency. There were no significant differences between two groups or two tasks. *Significant difference between two groups. **Significant difference between two tasks. ***Significant difference before and after video‐watching block.

Latency values (Figure 6) did not significantly differ between groups in either task. PD participants showed longer latencies in prosaccade tasks and slightly shorter latencies in antisaccade tasks, but neither reached significance. CVs followed the same pattern and were not significantly different between groups.

#### 3.1.5 Peak Velocity

**FIGURE 7:**
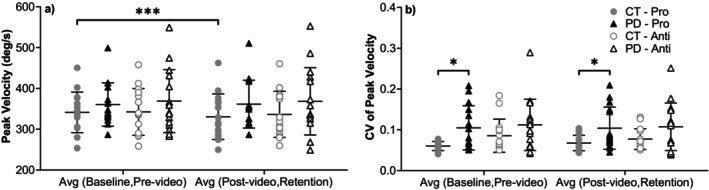
(a) Peak velocity. There were no significant differences between the two groups or two tasks in each group. In CT group, there were significant differences between the average of baseline and prevideo blocks and the average of postvideo and retention blocks (*p* = 0.035) of the prosaccade task. (b) CV of peak velocity. There were significant differences between the two groups in the prosaccade task of both the average of baseline and prevideo blocks (*p* = 0.021) and the average of postvideo and retention blocks (*p* = 0.037). *Significant difference between two groups. **Significant difference between two tasks. ***Significant difference before and after video‐watching block.

Peak velocity (Figure 7) tended to be higher in the PD group in both tasks, but differences were not statistically significant. CV of peak velocity, however, was significantly higher in the PD group for the prosaccade trials in both the average of baseline and prevideo blocks (U = 57, *p* = 0.021, $r_{\mathrm{rb}}$ = −0.493), and the average of postvideo and retention blocks (U = 62, *p* = 0.037, $r_{\mathrm{rb}}$ = −0.448). No significant differences were observed for antisaccade trials.

#### 3.1.6 Peak Acceleration

**FIGURE 8:**
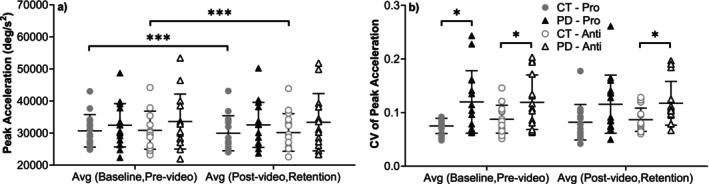
(a) Peak acceleration. There were no significant differences between two groups or two tasks in each group. Comparing before/after video‐watching blocks, in CT group, there were significant differences between the average of baseline and prevideo blocks and the average of postvideo and retention blocks in both pro‐/antisaccade tasks (*p* = 0.035, 0.048). (b) CV of peak acceleration. There were significant differences between two groups in the average of baseline and prevideo blocks (*p* = 0.037) of prosaccade task. In antisaccade task, differences reached significance in both the average of baseline and prevideo blocks (*p* = 0.043) and the average of postvideo and retention blocks (*p* = 0.023). *Significant difference between two groups. **Significant difference between two tasks. ***Significant difference before and after video‐watching block.

Peak acceleration (Figure 8) did not differ significantly between groups in either task. CV of acceleration was significantly higher in the PD group for the prosaccade task, in the average of baseline and prevideo blocks (U = 62, *p* = 0.037, $r_{\mathrm{rb}}$ = −0.448). For the antisaccade task, acceleration variability was significantly higher in PD in both the average of baseline and prevideo blocks (*t*(20.865) = 2.152, *p* = 0.043, Hedges' g = 0.72, 95% CI [−0.02, 1.46]) and the average of postvideo and retention blocks (U = 58, *p* = 0.023, $r_{\mathrm{rb}}$ = −0.484).

### 3.2 Task Differences (Pro‐ vs. Antisaccades)

Antisaccade error rates were significantly higher than prosaccade error rates in both groups (controls: *p* ≤ 0.007, PD: *p* ≤ 0.022, effect sizes between 0.73 and 1.13).

Peak velocity/amplitude did not differ significantly between tasks, although antisaccade trials showed greater variability. For controls, the CV difference reached significance in the average of postvideo and retention blocks (*t*(14) = 3.352, *p* = 0.005, Hedges' g = 0.82, 95% CI [0.23, 1.41]). No significant differences were observed in PD.

Gain was significantly higher in antisaccade tasks than prosaccade tasks in both groups (*p* < 0.001, effect sizes between 1 and 2). In contrast, gain variability was significantly higher in prosaccade tasks (*p* ≤ 0.001, effect sizes between 0.87 and 1.31).

Latency was significantly longer for antisaccade tasks than prosaccade tasks in both groups (*p* < 0.001, effect sizes between 1 and 1.82). Latency variability differed by block and group but did not reach significance.

Peak velocity variability tended to be higher in antisaccade tasks, with a near‐significant difference in controls in the average of postvideo and retention blocks (*t*(14) = 2.124, *p* = 0.052, Hedges' g = 0.49, 95% CI [−0.05, 1.03]).

Peak acceleration and its variability did not differ significantly between tasks.

### 3.3 Fixation Metrics During Video Viewing

**FIGURE 9:**
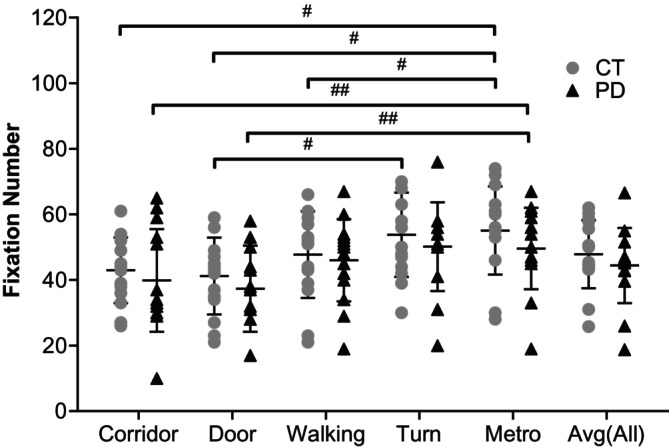
Number of fixations. There were no significant differences between two groups. In CT group, there were significant differences between ‘Metro’ and ‘Corridor’, ‘Door’ and ‘Walking’ videos (*p* = 0.005, < 0.001, 0.046). Also, ‘Turn’ and ‘Door’ videos were significantly different in CT group (*p* = 0.019). In PD group, there was significant difference between ‘Metro’ and ‘Corridor’ and ‘Door’ videos (*p* = 0.028, 0.006). ^#^Significant difference between two videos in CT group. ^##^Significant difference between two videos in PD group.

**FIGURE 10:**
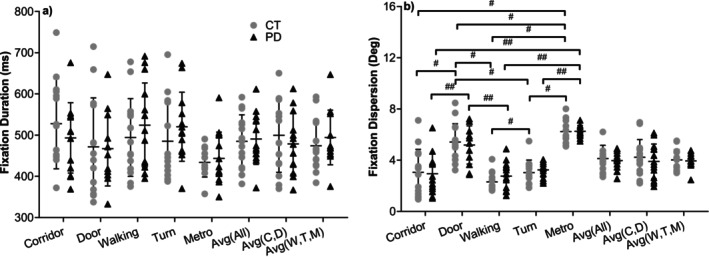
(a) Fixation duration. There were no significant differences between two groups or videos in each group. (b) Fixation dispersion. There were no significant differences between two groups. In CT group, there were significant differences between ‘Metro’ and ‘Corridor’, ‘Door’, ‘Walking’ and ‘Turn’ videos (*p* = 0.001, 0.041, 0.001 and 0.001). Also, there were significant differences between ‘Turn’ and ‘Door’ and ‘Walking’ videos (*p* = 0.002, 0.028) and ‘Door’ and ‘Corridor’ and ‘Walking’ videos (*p* = 0.001) in CT group. In PD group, there were significant differences between ‘Metro’ and ‘Corridor’, ‘Walking’ and ‘Turn’ videos (*p* < 0.001), between ‘Door’ and ‘Corridor’ and ‘Walking’ videos (*p* = 0.005, 0.013). ^#^Significant difference between two videos in CT group. ^##^Significant difference between two videos in PD group.

Fixation measures during video viewing (Figures 9, 10) showed no significant group differences in fixation count, fixation duration or fixation dispersion. The control group exhibited slightly higher fixation counts across videos; however, these differences did not reach statistical significance.

Video content influenced fixation patterns within each group. The ‘Metro’ video elicited the highest fixation counts, whereas the ‘Door’ and ‘Corridor’ videos elicited the fewest, with significant within‐group differences across video conditions. Fixation duration and fixation dispersion also differed across videos; however, group contrasts for individual videos or for grouped videos did not reach significance. Together, these results indicate that video content modulated fixation behaviour, but fixation‐based measures did not differentiate participants with PD from controls.

### 3.4 Pre‐ vs. Postvideo Changes in Saccade Metrics

No significant changes were observed in either group when comparing baseline versus prevideo or postvideo versus retention block, confirming stability across adjacent blocks.

When comparing the average of baseline and prevideo blocks (prevideo exposure blocks) with the average of postvideo and retention blocks (postvideo exposure blocks), significant changes were observed only in the control group. In controls, antisaccade peak velocity/amplitude decreased significantly (*t*(13) = 2.586, *p* = 0.023, Hedges' g = 0.15, 95% CI [−0.36, 0.66]), and both prosaccade gain and prosaccade peak velocity showed significant reductions (*t*(14) = 3.277, *p* = 0.007, Hedges' g = 0.82, 95% CI [0.23, 1.41]; and *p* = 0.035, $r_{\mathrm{rb}}$ = 0.6, 95% CI [0.42, 1.18], respectively). Antisaccade peak velocity demonstrated a marginal decrease (*t*(14) = 2.052, *p* = 0.059, Hedges' g = 0.51, 95% CI [−0.03, 1.05]). Peak acceleration decreased significantly in both pro‐ and antisaccade tasks (*p* = 0.035 and *p* = 0.048; $r_{\mathrm{rb}}$ = 0.6, 95% CI [0.42, 1.18]). No significant pre‐ to postvideo changes were observed in the PD group.

## 4 Discussion

### 4.1 Overview of Key Findings

The present study examined saccadic and fixation eye movement parameters in individuals with early‐ to midstage Parkinson's disease (PD) without FOG, assessed in the on‐medication state, compared with age‐ and sex‐matched neurologically healthy controls. By evaluating both traditional saccade metrics and trial‐to‐trial variability across reflexive (prosaccade), voluntary (antisaccade) and dynamic visual viewing conditions, we aimed to characterize oculomotor alterations that may be present prior to the emergence of gait freezing. Overall, the findings indicate that increased variability and reduced consistency of saccadic execution, rather than uniform changes in mean performance, most reliably differentiated the PD group. This pattern is consistent with contemporary views that PD‐related motor dysfunction may manifest as instability of movement output rather than simple slowing or hypometria, particularly in earlier disease stages (Pretegiani and Optican 2017; Antoniades and Spering 2023; Nemanich and Earhart 2016). These results should be interpreted considering the exploratory design, modest sample size and dopaminergic medication state.

### 4.2 Variability as a Prominent Feature of Oculomotor Control in Parkinson's Disease

Across saccade tasks, individuals with PD without FOG demonstrated significantly greater trial‐to‐trial variability in multiple parameters, including peak velocity, acceleration, gain and the peak velocity‐to‐amplitude ratio. This pattern was especially evident during prosaccade trials, where several variability measures reached statistical significance even when mean parameter values did not differ robustly between groups. Increased motor variability has been increasingly recognized as a characteristic feature of PD and may reflect instability within basal ganglia–cortical circuits involved in sensorimotor integration and movement scaling (Pretegiani and Optican 2017; Antoniades and Spering 2023; Nemanich and Earhart 2016).

Evidence from limb motor control, gait and oculomotor studies suggests that PD‐related dysfunction may be more sensitively captured by variability‐based measures than by average performance metrics alone, particularly in nonfreezing or early disease cohorts (Antoniades and Spering 2023; Nemanich and Earhart 2016). In this context, variability‐based saccade measures may provide complementary insight into oculomotor control in PD. However, despite their sensitivity at the group level, these measures should not be interpreted as diagnostic markers. Rather, they should be viewed as descriptive indicators that warrant further investigation in larger, hypothesis‐driven and longitudinal studies (Antoniades and Spering 2023).

### 4.3 Prosaccade Sensitivity and Task‐Dependent Effects

Although antisaccade performance has often been emphasized in PD due to its reliance on executive control and inhibitory processes (Briand et al. 1999; Chan et al. 2005; van Stockum et al. 2012; Walton et al. 2015), the most robust group differences in the present study emerged during prosaccade tasks. Specifically, prosaccade error rates and variability in the peak velocity‐to‐amplitude ratio were elevated in the PD group. Importantly, this finding does not imply that reflexive saccades are uniquely impaired in PD or that they are inherently superior for disease characterization. Instead, the relative sensitivity of prosaccade metrics in this study likely reflects task‐specific demands, analytic definitions and the medication state of participants. All PD participants were assessed while on dopaminergic medication, which has been shown to modulate reflexive saccade dynamics, including velocity, gain and variability (Stuart et al. 2019; Briand et al. 1999; Chan et al. 2005; Nemanich and Earhart 2016). Medication‐related effects may attenuate group differences in voluntary control tasks while simultaneously revealing variability in reflexive responses, helping to reconcile inconsistencies reported across previous studies of prosaccade performance in PD (Briand et al. 1999; Chan et al. 2005; van Stockum et al. 2012; Gorges et al. 2014; Nemanich and Earhart 2016).

### 4.4 Pro‐ Versus Antisaccade Performance

Consistent with prior literature, antisaccade trials were associated with higher error rates, longer latencies and greater gain than prosaccade trials in both groups, reflecting the increased cognitive demands of suppressing a reflexive response and generating a voluntary saccade (Antoniades et al. 2013; Briand et al. 1999; Chan et al. 2005; van Stockum et al. 2012). Gain values exceeding one during antisaccade trials suggest a tendency toward hypermetric responses under higher task complexity, a phenomenon previously reported in both healthy individuals and PD populations (Chan et al. 2005; van Stockum et al. 2012).

Variability in several kinematic parameters was also greater during antisaccade trials, particularly in the control group, consistent with increased task complexity and response uncertainty (Briand et al. 1999; Chan et al. 2005). Notably, gain variability was higher during prosaccade tasks in both groups, a pattern that contrasts with some prior reports (Nemanich and Earhart 2016). Differences in gain definition likely contribute to these discrepancies. In the present study, gain was defined relative to postcorrective fixation amplitude following the DEMoNS protocol (Nij Bijvank et al. 2018), an approach that accounts for potential inaccuracies in absolute eye position measurement and emphasizes methodological transparency.

### 4.5 Fixation Behaviour During Dynamic Video Viewing

Fixation metrics during naturalistic video viewing did not differ significantly between groups, although fixation behaviour was strongly modulated by video content in both PD and control participants. Videos depicting visually complex or socially salient environments elicited greater fixation counts and dispersion, whereas more constrained visual environments elicited fewer fixations. These findings are consistent with prior work demonstrating that fixation patterns during free‐viewing are highly sensitive to stimulus characteristics, scene complexity and attentional demands (Archibald et al. 2013; Munn et al. 2008; Andersson et al. 2017; Zhang et al. 2020).

The absence of robust group differences in fixation metrics may reflect compensatory strategies, task demands or limitations of fixation detection algorithms in dynamic scenes (Munn et al. 2008; Andersson et al. 2017). Importantly, studies examining fixation behaviour specifically in PD without FOG are limited. Graham et al. (2023), the only prior study to examine fixation during walking in nonfreezers, similarly reported no significant group differences in fixation duration. Future studies incorporating standardized video stimuli, additional gaze metrics and validated fixation detection approaches may improve sensitivity to disease‐related differences in visual exploration.

### 4.6 Contextual Effects of Video Viewing on Saccade Performance

When comparing saccade performance before and after video viewing, significant changes were observed only in the control group, whereas no reliable pre–postchanges were detected in the PD group. This finding should not be interpreted as evidence of impaired adaptability or learning in PD, as the present study did not directly assess adaptive mechanisms, reinforcement learning or neural plasticity. Rather, the absence of detectable changes in the PD group may reflect greater baseline variability in saccadic execution, which can obscure subtle context‐dependent modulation of oculomotor parameters (Antoniades and Spering 2023; Nemanich and Earhart 2016).

From a systems‐level perspective, increased motor variability in PD has been linked to altered basal ganglia–cortical signalling and reduced precision in movement scaling, particularly under conditions that require flexible updating of sensorimotor output (Pretegiani and Optican 2017; Antoniades and Spering 2023). In such circumstances, changes induced by contextual manipulation may fall within the range of intrinsic trial‐to‐trial variability, rendering them statistically undetectable at the group level. Thus, the lack of observed pre–postdifferences in the PD group may reflect limitations in signal detectability rather than an absence of context‐related modulation.

Previous work has demonstrated that visual context, environmental complexity and emotional salience can modulate oculomotor behaviour in neurologically healthy individuals, influencing saccade kinematics and visual exploration patterns (Archibald et al. 2013; Zhang et al. 2020). In contrast, increased baseline variability in PD may limit the detectability of such context‐driven effects when assessed using standard pre–postcomparisons. This interpretation is consistent with reports that PD‐related motor dysfunction often manifests as reduced consistency rather than uniform directional change, particularly in early or nonfreezing stages of the disease (Antoniades and Spering 2023; Nemanich and Earhart 2016).

Together, these findings underscore the importance of experimental paradigms that are explicitly designed to probe contextual modulation, adaptation or stress‐related influences on eye movements in PD. Approaches incorporating repeated exposures, parametric manipulation of stimulus salience or within‐trial adaptation metrics may be better suited to disentangle true context sensitivity from variability‐related masking effects in this population.

### 4.7 Limitations and Future Directions

Several limitations should be acknowledged. Although the sample size was modest, statistically significant effects with moderate‐to‐large effect sizes were observed for the primary comparisons, supported by acceptable reliability metrics. Replication in larger cohorts will be necessary for confirming generalizability and for characterizing inter‐individual variability within Parkinson's disease. All participants with Parkinson's disease were assessed in the on‐medication state, and medication dosage was not systematically controlled. While this approach enhances ecological validity by reflecting real‐world clinical functioning, dopaminergic therapy is known to influence saccade dynamics and may modulate the relative sensitivity of reflexive and voluntary oculomotor measures (Stuart et al. 2019; Briand et al. 1999; Chan et al. 2005; van Stockum et al. 2012; Gorges et al. 2014; Nemanich and Earhart 2016). Future studies directly comparing on‐ and off‐medication states will be essential for disentangling disease‐related effects from medication‐dependent modulation.

Certain analytic decisions, such as defining the main saccade based on peak velocity and applying task‐specific error thresholds, were intentionally selected to enhance robustness in a clinical population characterized by increased variability. Although these choices may limit direct comparability with studies using alternative definitions, they were applied consistently across groups and were grounded in established methodological frameworks (Nemanich and Earhart 2016; Nij Bijvank et al. 2018). Continued efforts toward analytic standardization will facilitate comparison across studies and strengthen cumulative inference.

Future research should incorporate larger cohorts, standardized analytic approaches, explicit comparison of Parkinson's disease subgroups with and without FOG and longitudinal designs. Such approaches will be particularly important for determining the stability, specificity and potential clinical relevance of variability‐based oculomotor measures across disease progression (Antoniades and Spering 2023).

## 5 Conclusion

The present findings demonstrate that oculomotor alterations in Parkinson's disease without FOG are most consistently expressed as increased trial‐to‐trial variability and reduced execution consistency, rather than uniform changes in mean saccade performance. Across tasks, variability‐based measures, particularly during prosaccade trials, were more sensitive to group differences than traditional kinematic metrics alone, underscoring the importance of examining stability of motor output when characterizing oculomotor control in Parkinson's disease. Differences between groups were most evident under reflexive saccade conditions and in the modulation of saccadic performance across task contexts. Importantly, the absence of detectable pre–postvideo effects in the Parkinson's disease group should not be interpreted as impaired adaptability, but rather as reflecting elevated baseline variability that may obscure subtle context‐dependent modulation. Fixation measures during dynamic viewing were strongly influenced by stimulus characteristics but did not reliably distinguish groups, highlighting the task‐specific nature of oculomotor sensitivity.

Together, these results emphasize the value of variability‐based oculomotor metrics as descriptive markers of altered sensorimotor control in Parkinson's disease. Future work using larger cohorts, longitudinal designs and systematic manipulation of medication state will be essential for determining the stability, specificity and potential clinical relevance of these measures, as well as for clarifying differences between Parkinson's disease subgroups.

## Author Contributions

F.S.D., M.S.F., S.B. and L.A. conceptualized the study, developed the methodology and designed the research approach. F.S.D. performed the experiments, collected data and conducted data analysis. M.S.F., S.B. and L.A. contributed to refining the data analysis methods and selecting appropriate analytical approaches. All authors (F.S.D., M.S.F., S.B. and L.A.) interpreted the data. F.S.D. and L.A. prepared the original draft and finalized the manuscript, while S.B. and M.S.F reviewed and provided critical revisions to the drafts.

## Conflicts of Interest

The authors declare no conflicts of interest.

## Data Availability

The data that support the findings of this study are available from the first author upon reasonable request, with approval of the corresponding author.
